# Case Report: Multiple electrolyte disturbance with severe neurological manifestations induced by chronic use of PPIs

**DOI:** 10.3389/fmed.2025.1646741

**Published:** 2025-09-17

**Authors:** Alexander Bertuccioli, Marco Cardinali, Francesco Di Pierro, Giordano Bruno Zonzini, Nicola Zerbinati, Maria Laura Tanda, Annalisa Belli, Chiara Maria Palazzi

**Affiliations:** ^1^Department of Biomolecular Sciences, University of Urbino Carlo Bo, Urbino, Italy; ^2^Microbiota International Clinical Society, Torino, Italy; ^3^Department of Internal Medicine, AST1, Pesaro e Urbino, Italy; ^4^Scientific & Research Department, Velleja Research, Milan, Italy; ^5^Department of Medicine and Technological Innovation, University of Insubria, Varese, Italy; ^6^Department of Medicine and Surgery, University of Insubria, Varese, Italy

**Keywords:** hypomagnesemia, proton pump inhibitors, electrolyte, polypharmacy, geriatric assessment, levetiracetam, epilepsy

## Abstract

**Background:**

Proton pump inhibitors (PPIs) are widely prescribed, especially in older adults with multiple comorbidities. However, their long-term use may lead to under-recognized adverse effects, including hypomagnesemia and related electrolyte disturbances, with potential neuromuscular and cognitive consequences.

**Case presentation:**

An 84-year-old man with several chronic conditions and prolonged PPI therapy presented with recurrent dysphagia, neuromuscular symptoms, and seizure-like episodes. Laboratory tests consistently showed hypokalemia and hypocalcemia, with intermittent hypomagnesemia, despite ongoing supplementation.

**Intervention:**

A comprehensive medication review led to the discontinuation of both pantoprazole and Levetiracetam. The Naranjo algorithm was used to assess causality, indicating a probable adverse drug reaction.

**Outcome:**

Following drug withdrawal, the patient experienced marked clinical improvement, with normalization of electrolyte levels. He no longer required supplementation and maintained stability through dietary management and adjustment of his antihypertensive therapy.

**Conclusion:**

This case highlights the dual iatrogenic role of PPIs and antiepileptics in causing persistent electrolyte imbalances. It emphasizes the need for regular medication reviews in elderly, polymedicated patients to prevent functional decline and promote recovery.

## 1 Introduction

Proton pump inhibitors (PPIs) are commonly prescribed for the treatment of peptic ulcer disease and gastroesophageal reflux disease (GERD), primarily by suppressing gastric acid secretion. Their protective effect against NSAID-related complications has contributed to their widespread use. However, concerns have emerged about the long-term safety of PPIs, with growing evidence linking chronic use to adverse effects involving the renal, cardiovascular, skeletal, pulmonary, and neurological systems (1). Among these, electrolyte disturbances, particularly hypomagnesemia, have gained growing attention (2–5). The most cited mechanism involves impaired intestinal magnesium absorption due to inhibition of transient receptor potential melastatin types 6 and 7 (TRPM6 and TRPM7) channels (6), as well as with reduced paracellular transport via downregulation of intestinal claudins (7, 8). Severe magnesium deficiency can lead to life-threatening complications such as cardiac arrhythmias, seizures, and secondary hypokalemia, hypocalcemia, and hyponatremia (6, 9). Several case reports describe these disturbances in the context of PPI use (3, 10). Here, we report a case of multisystem symptoms initially attributed to infective, neurological, and metabolic disease but ultimately linked to chronic PPI therapy.

## 2 Case description

This case report adheres to the CARE guidelines (11). Written informed consent was obtained from the patient for publication of this case report. Here we describe the case of a Caucasian male patient in his 80s. His medical history included a transient ischemic attack (1998), type 2 diabetes mellitus, dyslipidemia, hypertension, metabolic syndrome, and benign prostatic hyperplasia. Chronic medications included Nebivolol 5 mg, Canrenone 50 mg, acetylsalicylic acid 100 mg, Amlodipine 10 mg, Doxazosin 2 mg, Atorvastatin 20 mg, Metformin 500 mg three times daily, Pantoprazole 20 mg, and Dutasteride 0.5 mg.

In January 2022, after a first-ever seizure, he was diagnosed with multi-territorial ischemic encephalopathy with epileptogenic features, hypokalemia (2.5 mEq/L), and hypocalcemia (7.0 mg/dL); magnesium was not assessed. Levetiracetam 500 mg twice daily, Cholecalciferol 10,000 IU weekly, Calcium carbonate, Magnesium pidolate, and Potassium chloride were added to his regimen.

After initial stability, the patient developed worsening dysphagia and gastrointestinal symptoms leading to feculent vomiting—later attributed to suspected paralytic ileus—and was hospitalized again in March 2022. He was diagnosed with electrolyte imbalance (K 3.5 mEq/L, Mg 1.6 mg/dL, Ca 7.3 mg/dL) and chronic gastropathy. Pantoprazole was increased to 40 mg, and Canrenone to 50 mg twice daily.

Despite poor prognosis and functional decline, discontinuation of Levetiracetam resulted in progressive improvement in swallowing, ambulation, nutritional intake, and a good general recovery. However, the patient remained dependent on full-dose oral electrolyte supplementation and required periodic intravenous rehydration (Sodium chloride, Potassium chloride, Calcium chloride, Magnesium chloride, Sodium acetate, Sodium citrate).

Glycemic management was optimized: Metformin was replaced with Sitagliptin 100 mg in the morning and insulin Glargine 15 IU at bedtime. This clinical stability persisted until early 2025, when the patient experienced generalized seizures causing oral trauma and nearly choking on food, which required urgent care and hospitalization for pneumonia, chronic hypokalemia, and dehydration.

To facilitate reading, Table 1 presents the clinical information described in the text in a concise and chronological format, with particular attention to electrolyte values and key moments in therapeutic management.

**Table 1:** Clinical information presented in a concise and chronological format, highlighting electrolyte values and key moments in therapeutic management.

Date	Event/condition	Electrolytes (K/Mg/Ca) mg/dL	Intervention	Outcome
January 2022	Seizure, encephalopathy	2.5/n.d./7.0	Levetiracetam + supplementation	Partial response, persistent dysphagia
March 2022	Fecal vomiting, electrolyte imbalance	3.5/1.6/7.3	↑ Pantoprazole, ↑ Canrenone	Bedridden, worsening dysphagia
January 2025	Seizure-like episode, pneumonia	3.5/0.60/n.d.	Levetiracetam reintroduced	Dysphagia recurred
January 2025	Pneumonia resolution	4.94/1.73/n.d.	Discontinued antibiotics	persistence of neurological symptoms
February 2025	Therapy review	5.98/2.23/8.79	Discontinued Pantoprazole + Levetiracetam	Full recovery, normalized electrolytes

## 3 Diagnostic assessment

During the hospital evaluation, a complete laboratory panel was performed, including full blood count, renal and hepatic function tests, and electrolyte levels. A chest X-ray revealed consolidation in the right upper lung zone. These findings confirmed the diagnosis of early-stage pneumonia, hypokalemia (3.5 mg/dL), and initial dehydration. Despite the patient’s remote pathological history and the specific request from caregivers, magnesium and other electrolytes were not initially assessed, as they are not routinely measured in emergency department blood exams. Following the onset of neurological symptoms (confusion, lethargy, and eventually a seizure), magnesium was finally tested and revealed severe hypomagnesemia (0.60 mg/dL).

After neurological evaluation, Levetiracetam was reintroduced, coinciding with a rapid relapse of dysphagia. The patient was treated with Levofloxacin 500 mg in the morning and Clarithromycin 500 mg every 12 h to be continued at home for 7 days, the patient was then discharged. Three days post-discharge, pneumonia worsened, and systemic symptoms (asthenia, anorexia, dysphagia) progressed. Repeat labs showed leukocytosis (white blood cell count—WBC 30.46 × 10^9^/L–30.460/μL), C-reactive protein (CRP) 36.12 mg/L, and procalcitonin (PCT) 0.25 ng/mL.

### 3.1 Differential diagnosis

Antibiotic therapy with Ceftazidime 2 g every 8 h for 14 days and Azithromycin 500 mg daily for 6 days effectively resolved the infection; however, neurological and systemic symptoms persisted. Although nonspecific symptoms such as anorexia, asthenia, and nausea are common in elderly patients and can have multifactorial etiologies, a comprehensive laboratory workup is warranted. This should include a complete blood count with hemoglobin evaluation, thyroid-stimulating hormone (TSH), C-reactive protein (CRP), and a full serum electrolyte panel encompassing sodium, potassium, calcium, and magnesium. Differential diagnosis must consider anemia, infectious diseases (viral and bacterial), hypothyroidism, and rheumatologic disorders frequently diagnosed in the elderly, such as polymyalgia rheumatica, particularly in cases of acute or subacute onset of anorexia and asthenia. Subacute onset of nausea, absent evident causes like drug side effects or intestinal obstruction, especially when accompanied by anemia, warrants upper gastrointestinal endoscopy. Investigations revealed:

- Normal TSH and CRP levels
- Absence of active infection
- Negative brain CT scan for acute lesions
- Magnesium level re-evaluated, marginally below normal (1.73 mg/dL)

Following exclusion of common systemic and infectious causes, focus shifted toward electrolyte disturbances, with particular emphasis on magnesium homeostasis. Identification of hypomagnesemia necessitates thorough evaluation of the patient’s pharmacological history and potential etiologies. While prolonged fasting or chronic alcohol consumption may contribute to magnesium deficiency, impaired intestinal absorption and renal magnesium wasting represent the predominant causes. Chronic proton pump inhibitor therapy is a well-documented cause of reduced intestinal magnesium absorption. Renal magnesium loss, often secondary to loop or thiazide diuretics, should also be investigated through spot urine electrolyte measurements.

Additionally, hypercalcemia, commonly due to primary hyperparathyroidism, can reduce renal magnesium reabsorption and cause magnesium depletion (12). In this case, urine magnesium levels were not assessed; however, PPI-associated malabsorption was deemed the most probable etiology based on the patient’s medication history (13). In summary, the delayed recognition and assessment of hypomagnesemia likely contributed to the persistence and progression of clinical symptoms. This case underscores the critical importance of early, comprehensive electrolyte monitoring—including magnesium—in elderly patients presenting with nonspecific symptoms such as anorexia and fatigue, particularly in the context of chronic PPI use (12).

## 4 Therapeutic interventions

Correction of electrolyte imbalances is essential; however, identification and discontinuation of causative agents remain paramount. Withdrawal or dose reduction of proton pump inhibitors and potentially diuretics, medications known to impair intestinal absorption or increase renal magnesium excretion, is mandatory in cases of iatrogenic hypomagnesemia.

Intravenous magnesium supplementation is indicated in cases of severe electrolyte depletion or symptomatic electrolyte imbalance (see differential diagnosis section). Additionally, intravenous administration should be prioritized in patients with malabsorption syndromes, such as short bowel syndrome, or those unable to tolerate oral intake. Specifically, magnesium intravenous correction should be administered slowly over 12–24 h, using 4–8 g of intravenous Magnesium sulfate in unstable or symptomatic patients with relevant depletion. This slow administration is important because a high plasmatic magnesium peak concentration can reduce renal magnesium reabsorption, thereby limiting therapeutic efficacy. For the same reason, in asymptomatic patients able to tolerate oral supplements (requiring 240–1,000 mg/day of elemental magnesium), extended-release formulations such as Magnesium L-lactate or Magnesium chloride are preferred. These formulations also help minimize common adverse effects like bloating and diarrhea, which are frequently associated with magnesium oxide supplements.

Given that magnesium is predominantly stored intracellularly, supplementation should continue for several days following normalization of plasma magnesium levels.

Caution is warranted in patients with acute or chronic renal impairment, as impaired renal magnesium clearance may render treatment outcomes unpredictable. In this population, supplementation should be reserved for cases of severe hypomagnesemia, with close monitoring of serum magnesium levels following administration (14).

## 5 Outcomes and follow-up

Given the absence of focal neurological signs and the recurrence of severe dysphagia shortly after the reintroduction of Levetiracetam, the antiepileptic drug was discontinued again. Within 2 weeks, a complete resolution of dysphagia was observed. Strongly suggesting a causal role. Although Levetiracetam has been studied in post-stroke dysphagia rehabilitation (15), it is known to cause somnolence and reduced alertness, sometimes to a degree requiring discontinuation of therapy (16, 17), which may impair neuromuscular coordination involved in swallowing.

A comprehensive re-evaluation of the patient’s pharmacologic history and laboratory trends, considering literature associating long-term PPI therapy with impaired magnesium absorption, prompted the discontinuation of Pantoprazole. Hypomagnesemia has been reported in up to 36% of long-term PPI users (use longer than 6 months), with higher prevalence in elderly and polymedicated populations (18). Serum electrolytes were reassessed 10 days later: potassium levels increased significantly to 5.98 mg/dL without external supplementation, while serum magnesium (2.23 mg/dL) and calcium (8.79 mg/dL) normalized. The spontaneous correction in the absence of intravenous or oral supplementation, together with the lack of diuretics and despite no assessment of urinary magnesium excretion, supports a causal link between chronic PPI use and a multifactorial malabsorptive state, most likely of intestinal origin (19). Although urinary magnesium excretion was not measured in this case, the normalization of serum magnesium levels after discontinuation of Pantoprazole, despite the absence of supplementation or diuretic use, argues strongly in favor of a mechanism driven by impaired intestinal absorption rather than renal wasting. In frail, polymedicated elderly patients, routine measurement of urinary magnesium is not always feasible, and treatment decisions often rely on indirect clinical indicators, such as spontaneous correction after drug withdrawal. This pragmatic approach is particularly relevant when functional recovery parallels biochemical improvement.

According to the Naranjo Adverse Drug Reaction Probability Scale (20), assessed during a home visit by a personal physician, both Levetiracetam and Pantoprazole scored 7 (Tables 2, 3), indicating a probable adverse drug reaction. The positive dechallenge, temporal relationship, and partial correction of serum magnesium (1.73 mg/dL) and calcium (8.79 mg/dL) in the absence of supplementation further support a causal link with a multifactorial malabsorptive state. For Pantoprazole, temporal correlation, symptom improvement upon discontinuation, and the absence of alternative etiologies supported this classification, especially considering that clinical and biochemical recovery occurred despite the absence of supplementation or while maintaining the same supplementation regimen. Similarly, the rapid resolution of neuromuscular symptoms following Levetiracetam withdrawal, repeatedly observed after re-exposure, reinforced a pharmacological origin of the dysphagia and cognitive slowing. Although the scale does not replace clinical judgment, it remains a validated and widely used tool for assessing ADR likelihood in complex patients.

**Table 2:** Naranjo adverse drug reaction probability scale applied to Levetiracetam.

Question	Answer	Score	Justification
1. Are there previous conclusive reports on this reaction (e.g., to Levetiracetam)?	Yes	+1	Documented reports exist on neuromuscular and neuropsychiatric adverse effects.
2. Did the adverse event appear after the suspected drug (Levetiracetam) was administered?	Yes	+2	Symptoms developed shortly after Levetiracetam was started.
3. Did the adverse reaction improve when the drug was discontinued or a specific antagonist was given?	Yes	+1	Clinical improvement was observed after discontinuation.
4. Did the adverse reaction reappear when the drug was readministered?	Yes	+2	Symptoms recurred after reintroduction.
5. Are there alternative causes (other than Levetiracetam) that could have caused the reaction?	Not known	0	No clear alternative cause identified.
6. Did the reaction reappear when a placebo was given?	Not known	0	No placebo challenge was performed.
7. Was the drug detected in any body fluid in toxic concentrations?	Not known	0	No serum levels available.
8. Was the reaction more severe when the dose was increased or less severe when the dose was decreased?	Yes	+1	Symptom severity was dose-dependent.
9. Did the patient have a similar reaction to the same or similar drugs in any previous exposure?	No	0	No history of similar reaction.
10. Was the adverse event confirmed by any objective evidence (e.g., laboratory tests, imaging, EMG)?	Not known	0	No definitive objective evidence documented.

Total score: 7 (Probable ADR).

**Table 3:** Naranjo adverse drug reaction probability scale applied to Pantoprazole.

Question	Answer	Score	Justification
1. Are there previous conclusive reports on this reaction (e.g., to Pantoprazole)?	Yes	+1	There are documented cases of hypomagnesemia and neuromuscular effects associated with PPIs.
2. Did the adverse event appear after the suspected drug (Pantoprazole) was administered?	Yes	+2	Onset of symptoms followed the initiation of Pantoprazole.
3. Did the adverse reaction improve when the drug was discontinued or a specific antagonist was given?	Yes	+1	Clinical improvement and correction of magnesium levels occurred after discontinuation.
4. Did the adverse reaction reappear when the drug was readministered?	Not known	0	The drug was not reintroduced.
5. Are there alternative causes (other than Pantoprazole) that could have caused the reaction?	No	+2	Other causes of hypomagnesemia were excluded.
6. Did the reaction reappear when a placebo was given?	Not known	0	No placebo challenge was conducted.
7. Was the drug detected in any body fluid in toxic concentrations?	No	0	No toxic serum levels reported or available.
8. Was the reaction more severe when the dose was increased or less severe when the dose was decreased?	Not known	0	No dose adjustment information available.
9. Did the patient have a similar reaction to the same or similar drugs in any previous exposure?	No	0	No prior similar reaction documented.
10. Was the adverse event confirmed by any objective evidence (e.g., laboratory tests, imaging, EMG)?	Yes	+1	Hypomagnesemia confirmed through serum magnesium levels and related neuromuscular symptoms.

Total score: 7 (Probable ADR).

At the time the supplementation was stopped, the patient remained biochemically stable with dietary management alone. Antihypertensive therapy was optimized: Amlodipine was discontinued, Canrenone reduced to 25 mg, and Nebivolol replaced with Bisoprolol 2.5 mg. Functionally, the patient experienced significant clinical improvement. Dysphagia resolved completely, oral intake normalized, and independent ambulation was regained. Over the following 6 weeks, laboratory monitoring confirmed sustained normalization of serum magnesium, calcium, and potassium levels. No further episodes of seizure-like activity, neuromuscular dysfunction, or infectious complications were reported. The patient resumed daily activities with minimal caregiver support and maintained excellent adherence to the updated pharmacological regimen. The core clinical findings in this case were persistent hypokalemia, hypocalcemia, and hypomagnesemia, despite ongoing oral and intravenous supplementation. These abnormalities correlated with neuromuscular dysfunction and cognitive symptoms. Therapeutic discontinuation of Levetiracetam and Pantoprazole, both strongly implicated through the Naranjo algorithm, led to full and sustained correction of electrolyte levels and complete resolution of symptoms, without the need for further supplementation. This rapid and spontaneous biochemical normalization following drug withdrawal underscores the importance of targeted deprescribing in complex geriatric cases. This case advocates for routine monitoring of serum magnesium in elderly patients under chronic PPI therapy, especially in the presence of unexplained neuromuscular or gastrointestinal symptoms (21).

## 6 Discussion

This case illustrates how chronic use of Pantoprazole in elderly, multimorbid patients can lead to electrolyte imbalances, specifically hypomagnesemia, hypokalemia, and hypocalcemia, contributing to neurological and gastrointestinal symptoms.

In this case, chronic PPI exposure plausibly precipitated hypomagnesaemia with secondary hypokalaemia, a dyselectrolytaemia known to lower neural stability and to worsen lethargy and weakness, symptoms classically reported in hypomagnesaemia, thereby narrowing the tolerability window of Levetiracetam for CNS adverse effects such as somnolence and reduced alertness (22–24). Biophysically, Mg^2+^ provides a voltage-dependent block of NMDA receptors; deficiency removes this brake, promoting glutamatergic “noise” and hyperexcitability in brainstem–cortical arousal networks. In that excitatory milieu, SV2A-mediated presynaptic dampening by levetiracetam can more readily manifest as sedation (net suppression of ascending arousal when the system is already destabilised) (25, 26). Regarding dysphagia, swallowing is orchestrated by a Central Pattern Generator (CPG) in the medulla oblongata, located primarily in the nucleus tractus solitarius and nucleus ambiguus. This CPG relies on glutamatergic/NMDA signalling; experimental stimulation of NMDA receptors in the nucleus tractus solitarius evokes swallow-like patterns, while antagonism suppresses them (27). Hypomagnesaemia, by disinhibiting NMDA channels, and hypokalaemia, by impairing skeletal muscle excitability, can both perturb this circuit and the bulbar–pharyngeal musculature, clinically yielding dysphagia, described in hypomagnesaemia (including cases of esophageal spasm/dysphagia) and in hypokalaemic paralysis with bulbar involvement (23, 28, 29). On such a substrate, levetiracetam’s broad presynaptic reduction of transmitter release at both excitatory and inhibitory terminals (via SV2A) may further depress the already unstable swallow CPG output, aggravating dysphagia until the drug is withdrawn (25, 27). Mechanistically coherent links in this patient are therefore: PPI → hypomagnesaemia (± hypokalaemia via renal K^+^ wasting) → NMDA disinhibition + reduced neuromuscular excitability → increased susceptibility to Levetiracetam’s CNS-depressant AEs (somnolence, reduced alertness) and to swallow-network dysfunction (dysphagia), with symptom resolution after levetiracetam discontinuation compatible with its known AE profile and with correction of the dyselectrolytaemia (23, 24).

This case is clinically relevant because it highlights a rarely recognized but highly impactful dual pharmacological cause of chronic electrolyte imbalance in older adults: the combined effect of Levetiracetam and Pantoprazole. While PPI-induced hypomagnesemia is increasingly reported, the coexistence of neuromuscular symptoms triggered by both agents, and the complete clinical and biochemical recovery following their discontinuation, provides a unique demonstration of their synergistic iatrogenic burden. This dual mechanism, rarely documented in the literature, reinforces the need for critical medication review in elderly patients with unexplained multisystem symptoms.

**Figure 1:**
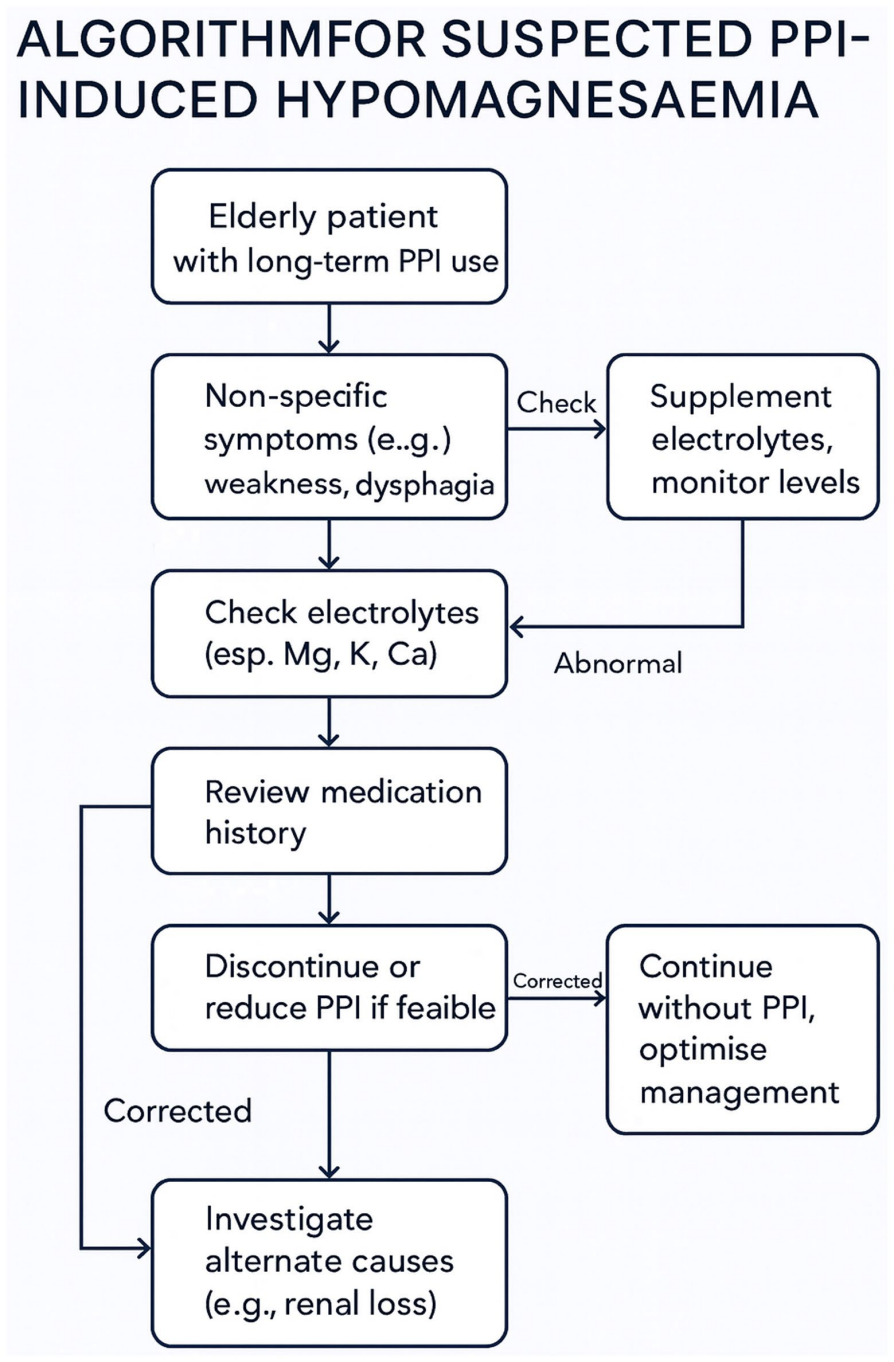
Algorithm for the management of suspected PPI-induced hypomagnesaemia.

**Figure 2:**
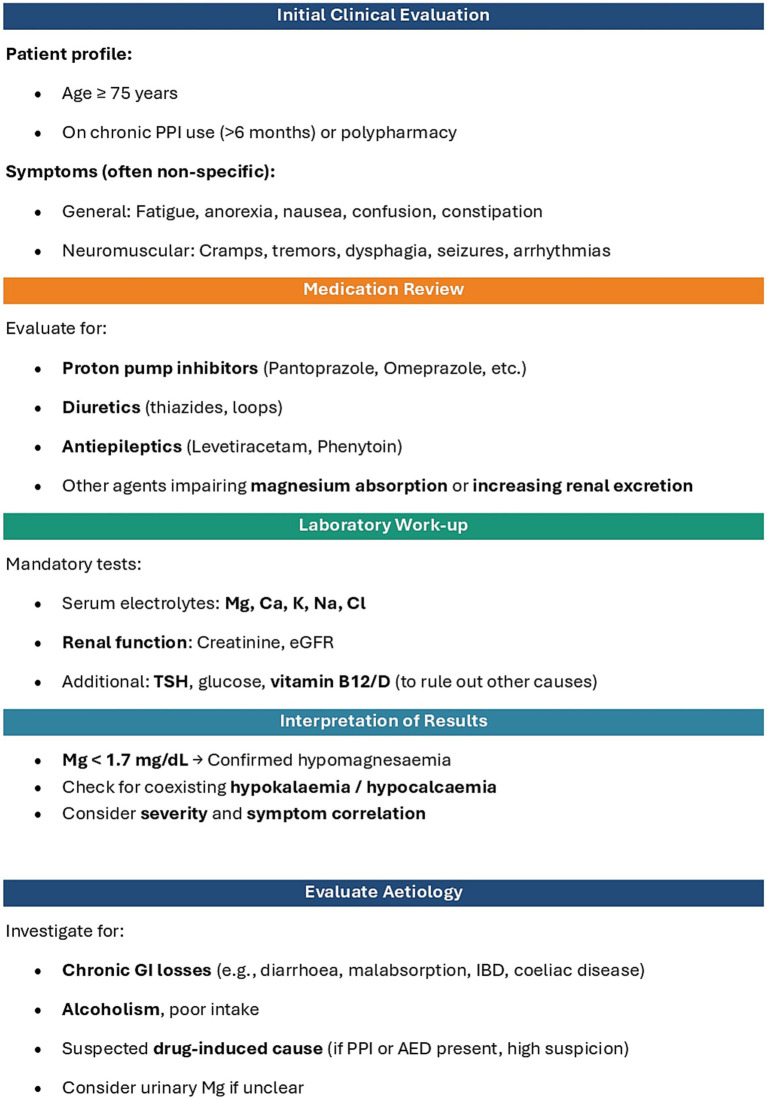
Clinical approach to suspected drug-induced hypomagnesaemia.

Only withdrawal of both Pantoprazole and Levetiracetam led to full clinical recovery. The constellation of symptoms, ranging from gastrointestinal dysmotility and neuromuscular dysfunction to seizure-like episodes, resolved only after the withdrawal of the offending agents (30–32). The prevalence and insidious nature of such imbalances make them a frequent yet underdiagnosed cause of functional decline in geriatric patients (18). Due to the diagnostic challenges associated with intestinal malabsorption and resulting hypomagnesemia, often triggering a cascade of symptoms that can worsen an already fragile clinical condition, Figure 1 presents a multidisciplinary action flowchart. This approach may be applicable to all patients over the age of 75 who are on chronic proton pump inhibitor therapy (lasting more than 6 months) or undergoing polypharmacological treatment. This flowchart is described point by point in Figure 2.

The recurrence of dysphagia following Levetiracetam reintroduction emphasizes the importance of individual drug assessment in geriatric care. This case underlines the often-overlooked role of drug-induced electrolyte disturbances and reinforces the importance of regular medication reviews.

This case underscores the clinical relevance of subtle electrolyte imbalances and their often-overlooked iatrogenic origins. It also reinforces the value of regular therapeutic reassessment, individualized deprescribing strategies, and a multidisciplinary approach in the management of complex geriatric patients. Periodic review of chronic medication regimens, including non-essential prophylactic drugs such as PPIs, should be considered standard practice in elderly care. This article highlights the importance of monitoring micronutrients, particularly magnesium, in elderly patients on chronic PPI therapy, to prevent the worsening of complex clinical conditions and improve quality of life. Timely recognition and correction of drug-induced adverse effects can significantly improve functional outcomes and quality of life, even in patients initially considered to have a poor prognosis.

The main limitations of this report are related to the recent onset of the case, which currently limits follow-up, and to the fact that some investigations to exclude malabsorption or renal losses were not performed in consideration of the patient’s age and the resolution of symptoms. Furthermore, the patient was managed by at least three different teams, which limited the possibility of a fully coordinated approach.

Below are some practical recommendations for clinicians:

- Monitor magnesium levels in patients on prolonged PPI therapy, especially if they experience cramps or weakness.
- Discontinue or reduce PPI use if hypomagnesemia is detected.
- Supplement magnesium, if necessary, preferably after adjusting the therapy.
- Inform patients about possible side effects of PPIs and symptoms to report.
- Periodic review and potential adjustment of pharmacological therapy upon the appearance of new suggestive symptoms (with further investigation in the scientific literature in case of seemingly non-specific symptoms), before considering the introduction of additional medications.

## Data Availability

As the data could potentially identify the patient, no supplementary material has been provided. The data are available from the corresponding author upon request.
